# Acute Paraplegia as a Presentation of Aortic Saddle Embolism

**DOI:** 10.1155/2016/1250153

**Published:** 2016-10-16

**Authors:** Lisandro Irizarry, Anton Wray, Kim Guishard

**Affiliations:** Department of Emergency Medicine, Wyckoff Heights Medical Center, Brooklyn, NY 11237, USA

## Abstract

*Background*. Acute onset paraplegia has a myriad of causes most often of a nonvascular origin. Vascular etiologies are infrequent causes and most often associated with postsurgical complications.* Objective*. To describe the occurrence and possible mechanism for aortic saddle embolism as a rare cause of acute paraplegia.* Case Report*. Described is a case of a 46-year-old female who presented with the sudden onset of nontraumatic low back pain with rapidly progressive paraplegia which was subsequently determined to be of vascular origin.

## 1. Introduction

Low back pain is a common presenting complaint comprising approximately 3% of all emergency department visits, the majority of which are the result of injuries sustained at home [1]. A small percentage of similar individuals presenting in a primary care setting develop concomitant neurologic symptoms [2]. The onset of nontraumatic paraplegia represents a significant event for the patient and an emergent diagnostic challenge for the clinician. The consequence for the patient from either a delay or misdiagnosis can be catastrophic. The etiology of paraplegia is broad and includes disorders of the spinal cord which encompass external compression, infection, ischemia, and other nonspinal disorders. A structured approach to early intervention and management is imperative if neurologic function is to be preserved. Presented is a rare case of aortic saddle embolism associated with the sudden onset of low back pain and acute onset paraplegia.

## 2. Case Report

A 46-year-old female presented to the emergency department (ED) with a complaint of the sudden onset of low back pain. The symptoms began shortly prior to arrival when the patient experienced sudden onset of severe pain upon standing from a seated position. The pain was rated 10/10 and was localized across the low lumbar area with radiation to both legs. The patient took no analgesics and self-transported to the ED.

On ED presentation, the patient was noted to be unable to ambulate secondary to pain. Her initial vital signs were as follows: blood pressure of 177/99; heart rate of 88 beats per minute and regular; respiratory rate of 18 breaths per minute with an oxygenation saturation of 99% on room air; and an oral temperature of 99°F. The patient was an obese female with a history of untreated pernicious anemia and significant smoking history. The patient had not seen a physician in more than three years and had no recent illness or injuries. Examination revealed diffuse lumbar tenderness with normal lower extremity strength and gross sensation. Detailed sensory examination could not be undertaken due to patient distress at the time of evaluation. Posterior tibial and dorsalis pedis pulses were noted to be present bilaterally. Reflexes were found to be 2+ symmetrically with no evidence of saddle anesthesia. Shortly after presentation, the patient was noted to have urinated on herself because of a sense of “urgency.” Given this new finding, it was decided to immediately perform a noncontrast lumbar CT which showed no evidence of acute abnormalities. The patient was provided analgesics and a decision was made to monitor pain response.

**Figure 1:**
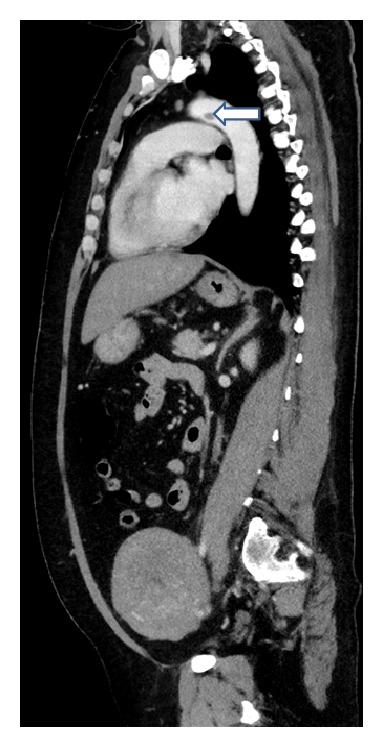
Chest computed tomography demonstrates thrombus (arrow) in the transverse aorta (sagittal view).

**Figure 2:**
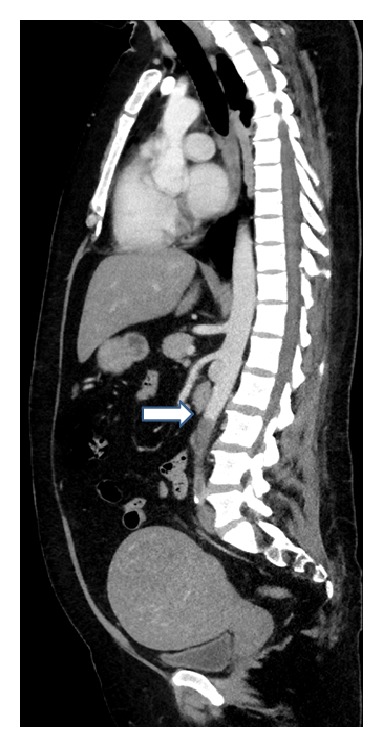
Abdominal computed tomography sagittal view demonstrates embolus (arrow) in the distal aorta to the level of the third lumbar vertebra.

Forty-five minutes after the analgesics were given, the patient experienced new onset of right lower extremity weakness. An emergent lumbar MRI was performed which showed no acute abnormalities. The patient continued to complain of severe back pain and progressively worsening lower extremity weakness which now included both lower extremities with the new onset of paraesthesias. Reexamination revealed pallor to the lower extremities, nonpalpable distal pulses, and decreased temperature to touch with near complete paraplegia. Concern for a vascular etiology prompted the performance of a chest and abdominal CT with contrast which revealed the near complete occlusion of the distal aorta with a saddle embolism which extended to the level of the third lumbar vertebra (Figures 1 and 2). A focal area of prominent plaque projecting into the aortic lumen at the level of the aortic arch was considered to be the potential source of the occlusive embolus. The patient was immediately taken to the operating room and underwent bilateral transfemoral arthrotomies with satisfactory retrieval of clots from the aorta and iliacs. Postprocedural bilateral angiograms showed superficial femoral, popliteal, and distal vessels with good runoff to the feet. Postprocedural transesophageal echocardiography revealed no clear cardiac source of the embolus. The patient's hospital course including a workup for a hypercoagulable state revealed no clear etiology for embolus formation. The patient experienced rapid improvement in clinical symptoms with mild residual right lower weakness, was provided aspirin and Plavix, and was ambulatory at discharge with the assistance of a walker.

## 3. Discussion

The onset of nontraumatic paraplegia represents an acute neurologic emergency requiring rapid recognition and management. The presentation is often not as clinically obvious as those of traumatic etiology; therefore a high index of clinical suspicion is needed to rapidly assess the various possible etiologies. Etiologies include vascular, inflammatory, space occupying lesions, and nonspinal disorders (see the following list).

*Differential Causes of Nontraumatic Paraplegia*

1. Extramedullary
  - Disc prolapse
  - Neoplasms
  - Epidural abscess
  - Epidural hematoma
  - Dural arteriovenous malformation
2. Intramedullary
  - Ischemia
    - Atherosclerosis
    - Vasculitis
    - Thrombosis/embolism
    - Vascular compression due to mass/operative complication
    - Decompression sickness
  - Myelitis
    - Viral
    - Bacteria
    - Parasitic
    - Parainfection
  - Demyelinating
    - Multiple sclerosis
    - Acute disseminated encephalomyelitis
3. Nonspinal disorders
  - Guillain-Barre syndrome
  - Hyperkalemic or hypokalemic paralysis
  - Tick paralysis

A structured approach to appropriately address each of the many causes may help the practitioner arrive at an accurate diagnosis thereby mitigating significant and permanent neurologic sequelae [3]. The initial step in the diagnostic evaluation of acute focal nontraumatic neurologic deficits is to rule out a compressive process through the performance of spinal MRI preferably with gadolinium contrast. If evidence of external spinal compression is found, emergent neurosurgical consultation is warranted. If no acute pathology is demonstrated, consideration should be given to vascular in addition to inflammatory and noninflammatory causes. It should be noted that patients with rapidly progressive findings over the course of a few hours should be presumed to have an ischemic etiology of possible vascular origin. As such, early consideration of possible causes is paramount to ensure maximal neurologic recovery. In consideration of inflammatory causes, lumbar puncture is performed with analysis of the cerebrospinal fluid for cell count, protein, and glucose levels, in addition to antibody synthesis and cytologic analysis. Positive findings should drive further diagnostic imaging to determine degree of demyelination of the neuraxis.

Vascular disorders resulting in decreased arterial flow to the spinal cord, although rare, must be expeditiously addressed. These disorders may result in abdominal aortic obstruction (AAO) precipitated from postoperative complications of aortic surgery including prolonged aortic clamp time, aortic dissection, thrombosis in situ of the aorta, or embolism to the distal aorta often from a primary cardiac source [4, 5]. Mortality associated with nonoperative management of AAO may be as high as 75%, while those with prompt surgical management continue to experience mortality rates of 20%–60% [6, 7]. Recent studies have demonstrated an evolving trend of nonprocedurally associated abdominal aortic obstruction (AAO) from predominantly embolic to predominantly thrombotic causes (76%) with an overall mortality of 34%. In addition, a significant portion of patients studied with thrombosis (40%) were demonstrated to have a potential hypercoagulable state including antiphospholipid antibody syndrome or other known malignancies [8–10]. Atherosclerotic aortoiliac disease may result in a subclinical presentation of intermittent claudication and buttock pain with a delay in acute findings due to the establishment of collateral blood flow [11]. These events rarely result in subtle and delayed presentations of vascular obstruction resulting in the erroneous evaluation and management of a neurologic etiology. Palpation of peripheral pulses as a single diagnostic method has previously been shown to be inaccurate in the assessment of adequate extremity perfusion [12]. The performance of the ankle-brachial index (ABI), easily performed and noted for its role in the evaluation of lower extremity peripheral arterial disease, has been noted to be of diagnostic utility in the assessment of acute vascular injury [13]. An ABI of <0.90 is of prognostic utility in the assessment of cardiovascular risk and functional impairment [14].

Paraplegia is a rare complication following aortic saddle embolus [15]. To fully appreciate the rarity of this complication of an embolic cause of AAO first reported nearly 35 years ago [16], one must understand the vascular supply to the distal spinal cord. The principle arterial supply to this region of the spinal cord is through the intrinsic vasculature provided via the greater radicular artery (GRA), also known as the Artery of Adamkiewicz. Obstruction to this vascular supply may result in an anterior spinal artery syndrome manifested by radicular pain, paraplegia, bladder and rectal tone dysfunction, and disruption of distal pain and temperature sensation. In approximately 85% of patients the GRA originates on the left side of the aorta at the level of seventh through twelfth thoracic vertebra [17]. In the remaining patients a low point of origin of the GRA may occur at the level of L3 (1.4%) or L4-L5 (0.2%) with the balance of the vascular supply provided by the anastomotic arterial ansa of the conus at L1–L5 supplied by the pelvic vasculature [18]. It is therefore unlikely that a distal aortic saddle embolism would result in spinal cord ischemia unless the obstruction is exceedingly long and completely occlusive or if the origin of the GRA is abnormally low [16]. Given that our patient had complete vascular obstruction to the level of the third lumbar vertebra accompanied with the rapid onset of a dense paraplegia it is highly likely that our patient has an abnormally low origin to the GRA. As there were no pre- or postvascular arteriograms performed, we were unable to confirm the origin of the patient's GRA.

## 4. Conclusion

The development of acute paraplegia represents a medical emergency of critical concern. Its diagnosis requires both an astute clinical acumen and use of appropriate diagnostic tools to enhance the possibility of a positive functional outcome. An expedited evaluation should follow a structured approach with the initial focus on an extrinsic cause for spinal cord compression diagnosed through the use of either computerized tomography or, if available, preferably, magnetic resonance imaging with contrast enhancement. Clinical findings suggestive of a vascular etiology may be further supported by the findings of an ankle-brachial index below 0.90. Such findings may warrant further evaluation through the performance of a contrast enhanced abdominal CT and, if positive for vascular obstruction, require emergent surgical intervention. Once these conditions have been effectively eliminated, efforts should be directed at identifying potential inflammatory causes through CSF analysis.

Nonoperative abdominal aortic obstruction remains a relatively rare clinical presentation with a significant associated mortality rate. The etiology of this condition has evolved over time from embolic to thrombotic in nature likely due, in part, to the increasingly frequent use of pharmacologic agents meant to mitigate cardioembolic events and an increasing incidence of atherosclerotic cardiovascular disease. This patient's initial presentation was suggestive of spinal cord compression. Initial workup with MRI scan did not reveal a mechanical spinal cord compressive force as the cause of the paraplegia. This redirected the diagnostic focus towards a vascular etiology as a potential source of spinal cord ischemia. A saddle embolus resulting in AAO as a cause was found. Surgical intervention of the AAO resulted in the restoration of circulation to the spinal cord with near complete resolution of the patient's neurological symptoms.

## References

[1] Waterman B. R., Belmont P. J., Schoenfeld A. J. (2012). Low back pain in the United States: incidence and risk factors for presentation in the emergency setting. *Spine Journal*.

[2] Deyo R. A., Weinstein J. N. (2001). Low back pain. *The New England Journal of Medicine*.

[3] Román G. C. (2003). Proposed diagnostic criteria and nosology of acute transverse myelitis. *Neurology*.

[4] Rosenthal D., Southern Association for Vascular Surgery (1999). Spinal cord ischemia after abdominal aortic operation: is it preventable?. *Journal of Vascular Surgery*.

[5] Inamasu J., Hori S., Yokoyama M., Funabiki T., Aoki K., Aikawa N. (2000). Paraplegia caused by painless acute aortic dissection. *Spinal Cord*.

[6] Wong S. S. N., Roche-Nagle G., Oreopoulos G. (2013). Acute thrombosis of an abdominal aortic aneurysm presenting as cauda equina syndrome. *Journal of Vascular Surgery*.

[7] Yamamoto H., Yamamoto F., Tanaka F. (2011). Acute occlusion of the abdominal aorta with concomitant internal iliac artery occlusion. *Annals of Thoracic and Cardiovascular Surgery*.

[8] Dossa C. D., Shepard A. D., Reddy D. J. (1994). Acute aortic occlusion. A 40-year experience. *Archives of Surgery*.

[9] Crawford J. D., Perrone K. H., Wong V. W. (2014). A modern series of acute aortic occlusion. *Journal of Vascular Surgery*.

[10] Mcclain R. L., Pai S.-L. (2013). Acute aortic occlusion presenting as paraplegia: a catastrophic complication in an elective surgical patient. *A&A Case Reports*.

[11] Lai C.-H., Wang C.-H., Wu S.-Y., Shih H.-M. (2014). Aortoiliac occlusive disease presenting as sudden onset paraplegia. *Annals of Vascular Surgery*.

[12] Lundin M., Wiksten J.-P., Peräkylä T. (1999). Distal pulse palpation: is it reliable?. *World Journal of Surgery*.

[13] Mills W. J., Barei D. P., McNair P. (2004). The value of the ankle-brachial index for diagnosing arterial injury after knee dislocation: a prospective study. *Journal of Trauma—Injury, Infection & Critical Care*.

[14] Aboyans V., Criqui M. H., Abraham P. (2012). Measurement and interpretation of the Ankle-Brachial Index: a scientific statement from the American Heart Association. *Circulation*.

[15] Silver J. R., Buxton P. H. (1974). Spinal stroke. *Brain*.

[16] Dickson A. P., Lum S. K., Whyte A. S. (1984). Paraplegia following saddle embolism. *British Journal of Surgery*.

[17] Triantafyllopoulos G. K., Athanassacopoulos M., Maltezos C., Pneumaticos S. G. (2011). Acute infrarenal aortic thrombosis presenting with flaccid paraplegia. *Spine*.

[18] Shaw A., Anwar H., Targett J., Lafferty K. (2008). Cauda equina syndrome versus saddle embolism. *Annals of the Royal College of Surgeons of England*.

